# The Detection of Colorectal Cancer through Machine Learning-Based Breath Sensor Analysis

**DOI:** 10.3390/diagnostics13213355

**Published:** 2023-10-31

**Authors:** Inese Poļaka, Linda Mežmale, Linda Anarkulova, Elīna Kononova, Ilona Vilkoite, Viktors Veliks, Anna Marija Ļeščinska, Ilmārs Stonāns, Andrejs Pčolkins, Ivars Tolmanis, Gidi Shani, Hossam Haick, Jan Mitrovics, Johannes Glöckler, Boris Mizaikoff, Mārcis Leja

**Affiliations:** 1 Institute of Clinical and Preventive Medicine, University of Latvia, LV-1586 Riga, Latvia; inese.polaka@lu.lv (I.P.); linda.anarkulova@gmail.com (L.A.); elina.kononova@lu.lv (E.K.); viktors.veliks@lu.lv (V.V.); anna_marija.lescinska@lu.lv (A.M.Ļ.); ilmars.stonans@lu.lv (I.S.); andrejs.pcholkins@gmail.com (A.P.); marcis.leja@lu.lv (M.L.); 2 Department of Modelling and Simulation, Riga Technical University, LV-1048 Riga, Latvia; 3 Faculty of Medicine, University of Latvia, LV-1586 Riga, Latvia; 4 Riga East University Hospital, LV-1038 Riga, Latvia; 5 Faculty of Residency, Riga Stradins University, LV-1007 Riga, Latvia; 6 Health Centre 4, LV-1012 Riga, Latvia; ilona153@inbox.lv; 7 Liepaja Regional Hospital, LV-3414 Liepaja, Latvia; 8 Faculty of Medicine, Riga Stradins University, LV-1007 Riga, Latvia; ivars.tolmanis@gastrocentrs.lv; 9 Department of Doctoral Studies, Riga Stradins University, LV-1007 Riga, Latvia; 10 Digestive Diseases Centre GASTRO, LV-1079 Riga, Latvia; 11 Laboratory for Nanomaterial-Based Devices, Technion—Israel Institute of Technology, Haifa 3200003, Israel; gidishani@gmail.com (G.S.); hhossam@technion.ac.il (H.H.); 12 JLM Innovation GmbH, D-72070 Tübingen, Germany; jan.mitrovics@jlm-innovation.de; 13 Institute of Analytical and Bioanalytical Chemistry, Ulm University, 89081 Ulm, Germany; johannes.gloeckler@uni-ulm.de (J.G.); boris.mizaikoff@hahn-schickard.de (B.M.); 14 Hahn-Schickard, 89077 Ulm, Germany

**Keywords:** colorectal cancer, sensors, screening, breath analyzer, volatile organic compounds, machine learning

## Abstract

Colorectal cancer (CRC) is the third most common malignancy and the second most common cause of cancer-related deaths worldwide. While CRC screening is already part of organized programs in many countries, there remains a need for improved screening tools. In recent years, a potential approach for cancer diagnosis has emerged via the analysis of volatile organic compounds (VOCs) using sensor technologies. The main goal of this study was to demonstrate and evaluate the diagnostic potential of a table-top breath analyzer for detecting CRC. Breath sampling was conducted and CRC vs. non-cancer groups (105 patients with CRC, 186 non-cancer subjects) were included in analysis. The obtained data were analyzed using supervised machine learning methods (i.e., Random Forest, C4.5, Artificial Neural Network, and Naïve Bayes). Superior accuracy was achieved using Random Forest and Evolutionary Search for Features (79.3%, sensitivity 53.3%, specificity 93.0%, AUC ROC 0.734), and Artificial Neural Networks and Greedy Search for Features (78.2%, sensitivity 43.3%, specificity 96.5%, AUC ROC 0.735). Our results confirm the potential of the developed breath analyzer as a promising tool for identifying and categorizing CRC within a point-of-care clinical context. The combination of MOX sensors provided promising results in distinguishing healthy vs. diseased breath samples. Its capacity for rapid, non-invasive, and targeted CRC detection suggests encouraging prospects for future clinical screening applications.

## 1. Introduction

Colorectal cancer (CRC) is the third most common malignancy and the second most common cause of cancer-related deaths worldwide [1]. By 2040, the burden of CRC is expected to increase to 3.2 million new cases (a 63% increase) and 1.6 million deaths (a 73% increase) per year [2].

Since CRC lacks specific symptoms in its early stages, early detection becomes a critical factor in preventing metastases, reducing mortality, and improving future life expectancy and quality [3]. In many developed countries, screening programs are considered fundamental public health services aimed at detecting precancerous lesions in the colon and identifying CRC in its early stages [4].

Colonoscopy is considered the gold standard tool for diagnosing CRC, as it allows the direct visualization and detection of colonic polyps or advanced neoplasia. It offers the advantage of performing a polypectomy or obtaining a biopsy for histological evaluation [5]. However, there are downsides to using colonoscopy as the primary screening method. It is an invasive procedure that demands bowel preparation and sedation, carries risks of bleeding and perforation, and is time-consuming, costly, and often associated with negative patient experiences [6,7].

An alternative to colonoscopy is computed tomography colonography (CTC), which is safe and minimally invasive. Nonetheless, CTC is also time-consuming and expensive. Additionally, it involves exposure to ionizing radiation and has a low sensitivity for detecting lesions less than 10 mm in size. Furthermore, it has a high false positive rate because it cannot differentiate between fecal material and polyps [8].

Non-invasive screening tools are also available, such as the fecal occult blood test (FOBT). However, recently, FOBT has been replaced by the fecal immunochemical test (FIT) due to its ability to provide a more specific detection of colorectal disease and reduce the occurrence of false positive results that can be seen with FOBT [9].

While CRC screening is already part of organized programs in many countries worldwide, there remains a need for improved screening tools. The ideal cancer screening tool should be noninvasive, safe, convenient, easily accessible, and inexpensive [10].

In recent years, a potential approach for cancer diagnosis has emerged via the analysis of volatile organic compounds (VOCs) emitted from various bodily fluids such as urine and blood, as well as exhaled breath and tissues [11,12,13]. VOCs are produced by biochemical processes within the body and constitute the human volatilome. This profile is influenced by exogenous sources such as diet, environment, and microbiota activity. Furthermore, cancer cells also modify the VOC profile, rendering VOC fingerprinting highly informative about the metabolic activity [14]. Most VOC analyses have focused on exhaled breath samples due to their noninvasive nature and ease of collection and analysis [15].

The commonly used technique for analyzing VOCs is gas chromatography-mass spectrometry (GC-MS). Even though GC-MS is considered the gold standard for detecting VOCs owing to its high sensitivity and specificity, the method is expensive, time-consuming, and laboratory-bound, and it requires highly trained personnel for analysis [16].

Research has shown that various types of cancer, including lung, colorectal, gastric, breast, and mesothelioma, can be detected via the VOC pattern [17,18]. 

During recent decades, researchers have invested significant efforts in exploring methods of artificial olfaction inspired by the mammalian nose, which senses and discriminates mixtures of organic compounds. ‘Artificial nose’ devices are based on integrated arrays of chemical sensors combined with pattern recognition methods, drawing inspiration from the olfactory system, and are frequently referred to as ‘electronic noses’ or e-noses. In clinical settings, cross-reactive sensors are mainly used. These sensors respond to a range of VOCs and are combined with pattern recognition methods to create a distinctive ‘breath fingerprint’ for each type of cancer [19,20]. In previous research, researchers used e-noses to detect gastrointestinal cancers in exhaled breath, yielding promising results to effectively distinguish between cancer patients and healthy individuals [18,21].

The main goal of the present study was to demonstrate and evaluate the diagnostic potential of table-top breath analyzers for detecting CRC. 

## 2. Materials and Methods

### 2.1. Study Recruitment Process

Patients with morphologically confirmed colorectal adenocarcinoma were included in this study before undergoing surgery. These patients had previously undergone colonoscopy due to health complaints. The non-cancer group comprised study subjects without adenocarcinoma and high-risk precancerous lesions; these patients had also undergone colonoscopy prior to the final study grouping, which was conducted after the morphological report. Before breath sampling, participants completed a medical and family history questionnaire, as well as a lifestyle questionnaire that included potential confounding factors.

Study participants were recruited from Riga East Clinical University Hospital, the Oncology center of Latvia, and the Digestive Diseases Centre GASTRO. Subjects aged 18 and older who could donate a breath sample and had provided signed consent forms were included in this study. To exclude confounding factors of other diseases, the following exclusion criteria were applied: other malignancies are currently active; neoadjuvant chemotherapy and radiation therapy have been started; acute conditions (the patient is scheduled for emergency surgery); previous bowel resection; chronic renal failure stage 4; type I diabetes; active bronchial asthma; inflammatory bowel diseases; patients who have undergone a complete bowel cleansing (started using a bowel cleansing agent). 

A total of 476 patients participated in this study, but the analysis included only those patients who did not experience an erroneous change in the sensor reading.

### 2.2. Study Group Description 

In total, 291 individuals were included in analysis, among whom 113 were male and 178 were female, with a median age of 63. Specifically, 105 patients with histologically confirmed colorectal adenocarcinoma were enrolled in this study, as well as 186 non-cancer subjects. Detailed clinical characteristics of the study subjects are provided in Table 1. 

### 2.3. Breath Analyzer and Sample Collection

Breath sampling was conducted in both the CRC and control groups using a table-top breath analyzer. This device was developed by JLM Innovation GmbH and held 73 sensors in total. The device consists of a breath capture module and a sensor chamber module. The breath capture module has a prolonged arm for easy exhalation from a sitting position when the device is placed on a table. The opening for exhalation is fitted with a disposable mouthpiece to allow for intensive patient flow. The breath capture module is connected to a heated sensor chamber that holds 73 sensors. The set of sensors consisted of both stable and commercially available sensors, as well as experimental sensors that were developed to be selective toward a wide range of VOCs in exhaled breath. The sensors were used to analyze the room air before a study participant exhaled into the device, and then the exhaled breath.

The room air or baseline measurement was taken for 60 s to determine the response of the sensors to the background VOCs found in the ambient air that could affect the breath exhaled by the participant. Afterward, the participants exhaled into the device and the exhaled breath was measured for 60 s.

The device was connected to a computer and the metadata and response curves of the sensors to the ambient air and the exhaled breath for each participant were recorded into .json files. The pre-processing of the signals included removal of faulty measurements and sensors, normalization against room air, applying median smoothing (window size was set to five time points) to remove noise, as well as feature extraction from the response curves.

To minimize the influence of potential confounding factors on exhaled breath analysis, participants were given specific instructions: they were asked to provide breath samples following an overnight fasting period, and abstain from smoking, consuming alcohol, chewing gum, and engaging in physical activity for a minimum of 2 h prior to the sampling. Additionally, participants were recommended to refrain from using perfume until after the sample collection.

Breath samples were collected within a specially allocated and sanitized room. This room remained devoid of chemicals, cleaning agents, medicines, solvents, and kitchen refuse. A consistent temperature was upheld to diminish the effects of external factors, encompassing potential impurities originating from the hospital surroundings. 

### 2.4. Data Pre-Processing

To remove any signals introduced by differences in room air composition at different times, the sensor response to breath (*x_i_*) was normalized against the last ten time points from the response to ambient air (*y_i_*):

$${x^{\prime }}_{i}=\frac{\bar{y_{i\;(last\;10\;points)}}-x_{i}}{\bar{y_{i\;(last\;10\;points)}}}$$

The analysis of the pre-processed data was to be carried out using supervised machine learning methods, solving a classification task. Several features were extracted from the sensor response curves of each sensor to use as input for machine learning model induction:

▪ Minimum value of the curve.
▪ Average value of the curve.
▪ Maximum value of the curve.
▪ Mean value of the last 10 time points to characterize the sensor response after saturation.
▪ Area under the curve calculated using the trapezoidal rule.

### 2.5. Classification

Given the complex nature of the relationships among the different sensors that characterize the breath fingerprint (consisting of multiple VOCs) and the outcome class, they should be analyzed together to create representations of these relationships or models that can incorporate complex relationships, such as machine learning models. However, the methods and models should not be too complex, given the available sample size; therefore, methods like deep learning were not applied. Instead, the features characterizing the response curve of each sensor were used to induce machine learning models, including Random Forest, C4.5, Neural Network, and Naïve Bayes

C4.5 is a classic decision tree induction algorithm that induces tree-based models from data based on Gain Ratio [22]. This algorithm also has a built-in feature selection that helps to significantly reduce the number of sensors and their features. In the process of model induction, the algorithm chooses a feature that is used for data splitting based on the largest Gain Ratio (normalized Information Gain) from splitting a data set *S* using feature *A*:

$$GainRatio=\frac{Entropy\left(S\right)-Entropy(S|A)}{-\sum_{i=1}^{n}\frac{N(A_{i})}{N(A)}log\frac{N(A_{i})}{N(A)}}$$

where *N(Ai)* is the number of records in the subset, feature *A* has the *i*-th value, and *N(A)* is the total number of records.

Random Forest [23] is another popular tree-based method that induces an ensemble of random trees that use voting to determine the class of a record. The trees are constructed by selecting random subsets of features for each split and choosing the best feature based on Information Gain. The resulting class is determined by voting weighted by the performance of each tree.

Artificial Neural Networks are another popular approach to build a classification model by creating a network of nodes that are activated based on previous knowledge. The Neural Network algorithm used in this paper creates a network with one hidden layer, with the number of nodes set to

$$\frac{N\left(attributes\right)+N(classes)}{2}$$

The algorithm uses backpropagation training and Broyden–Fletcher–Goldfarb–Shanno algorithm for optimization. An approximate version of the logistic function serves as the activation function in the hidden layer, and the sigmoid function is used in the output layer. To diversify the classification approaches, a probability-based algorithm (Naïve Bayes algorithm [24]) is used to classify the records. This algorithm uses Bayes’ theorem to determine the probability of each class for a record:

$$P\left(c_{i}|x\right)=\frac{P\left(x|c_{i}\right)\cdot P(c_{i})}{P(x)}$$

where $c_{i}$ is class value and *x* is vector of feature values.

### 2.6. Dimensionality Reduction

The response curve of each of the 73 sensors was characterized using five features, which would result in 365 features in total if all sensors worked all the time. This is a relatively large feature set for a sample of fewer than 300 cases, considering the possible noise and presence of non-informative features (e.g., some sensors may not be picking up any signal that points to cancer), and the optimal classifier could use only a fraction of these sensors. Therefore, for methods that do not have a built-in feature selection, other feature selection techniques were used. This included the wrapper approach that selects a feature subset based on the performance of classification models in the train set using evolutionary algorithm or greedy stepwise algorithm for search. The evolutionary algorithm creates random subsets that are evolved, maximizing accuracy, while Greedy Search performs stepwise search through the feature set to find the optimal feature subset. In this study, we used forward selection starting from an empty set until the addition of a feature started decreasing the result.

### 2.7. Experimental Setup

The data pre-processing step was carried out using R version 4.1.2. [25] and libraries. The prepared data were then used as input for machine learning model induction using algorithms from Weka version 3.8.3 [26]: weka.classifiers.trees.RandomForest for Random Forest, weka.classifiers.trees.J48 for C4.5, weka.classifiers.functions.MultilayerPerceptron for Neural Networks, and weka.classifiers.bayes.NaiveBayes for Naïve Bayes. The models were trained using 70% randomly chosen records (features of sensor responses to patient breaths) of the whole data set, and the rest of the data set was used for blind testing. 

## 3. Results

### 3.1. Data Pre-Processing 

The explorative analysis detected some defective sensors that malfunctioned in some measurements. Therefore, 17 sensors were removed from the analysis to eliminate any erroneous signals, leaving 56 sensors (280 features) for model induction.

### 3.2. Classification Results

In the initial data sets, the overall classification accuracy (see Table 2) was insufficient. For most classification methods, this was due to low sensitivity, except for the Naïve Bayes method, which showed a high sensitivity while demonstrating low performance to correctly detect controls. This means that most of the methods cannot capture the patterns necessary to correctly identify the breaths of cancer patients.

Another reason for this could be the class imbalance, with 105 cancer patients and 186 controls. Therefore, we investigated if the situation would change if the number of both classes in the data sets was equal by selecting a random subset of 105 controls. The results, presented in Table 3, show improved overall accuracy with a 5–19 percent point increase in sensitivity, albeit with some decline in specificity. For most of the methods, the AUC ROC also increased, meaning that the overall capability of the models to differentiate between the classes improved.

Another way to increase the accuracy that was considered in this study is dimensionality reduction. To remove potential noise, and uninformative and redundant features, we applied two different feature selection methods to reduce dimensionality (results for the full data set are given in Supplementary Materials, Tables S1 and S2) and also considered selecting only a subset of features based on their type—gold nanoparticle (GNP) sensor subset and metal oxide (MOX) sensor subsets (see the results in Supplementary information, Tables S3 and S4 for results in full GNP and MOX sets, respectively, and Tables S5–S8 for results in feature subsets of these sets). Overall, Greedy Search created smaller feature subsets where the methods showed higher accuracy, compared to the feature subsets found through Evolutionary Search.

While the overall accuracy of the models was higher in the full GNP sensor subset, the results for sensor subsets (discovered using feature selection methods) showed that the best result was obtained using the C4.5 method in the MOX sensor set, when the feature set was reduced using the Wrapper approach and Greedy Search (overall accuracy: 77.0%, sensitivity: 63,3%, specificity: 84.2%, AUC ROC: 0.759). The best results for the feature subsets of GNP and MOX sensor sets for each method are given in Table 4.

The best overall accuracy was achieved (see Table 5) using Random Forest and Evolutionary Search for Features (79.3%, sensitivity 53.3%, specificity 93.0%, AUC ROC 0.734), and Neural Networks and Greedy Search for Features (78.2%, sensitivity 43.3%, specificity 96.5%, AUC ROC 0.735). While C4.5 showed a slightly worse overall accuracy, this method showed the most acceptable sensitivity—63.3%—and a good specificity—84.2%. The ROC plots for these models are given in the Supplementary Materials.

## 4. Discussion

Over the last few decades, various breath sensor technologies have emerged and found application within controlled laboratory environments. Additionally, there have been documented instances of electronic olfaction systems being integrated with advanced data analytics techniques. Breath assessment holds great promise as a non-invasive method for early cancer detection. Recent advancements have highlighted its potential in the realm of gastrointestinal cancers, including CRC [16,27,28,29].

In this study, we evaluated the diagnostic performance of a tabletop breath sensor analyzer in detecting CRC patients. We assessed the specificity of the sensors alongside other performance metrics, such as accuracy, sensitivity, and AUC ROC. Among the various feature selection methods employed, the C4.5 method stood out by achieving a specificity of 84.2% for the MOX sensor set. This suggests the effectiveness of MOX sensors in distinguishing healthy breath samples. 

Furthermore, our study highlights that while other methods might have achieved higher overall accuracy, the C4.5 method managed to maintain a good balance between sensitivity—63.3%—and specificity—84.2% (AUC 0.759). This trade-off is crucial in CRC screening where false positives can lead to unnecessary further invasive testing, such as colonoscopy, which is time- and resource-consuming and leads to undue stress for individuals, a procedure that requires a lot of strict rules to comply [30,31]. Meanwhile, FIT tests are not ideal, due to their low sensitivity to detect early-stage, small-size, and proximally located neoplasms [32]. Hence, the sensor technology is currently of utmost interest [27,33].

Recent studies on the subject reveal a degree of fluctuation in specificity and sensitivity of sensor technologies. Ultimately, these studies strive for equilibrium readings to achieve a satisfactory level of accuracy. 

The study conducted by van Keulen et al. utilized e-nose technology to train models for detecting CRC and precursor lesions. In this study, initial AUC was measured at 0.76 for CRC, and during the blind validation phase, the AUC stood at 0.74 for CRC. The culmination of these efforts yielded final models for CRC with an AUC of 0.84, characterized by a sensitivity of 95% and specificity of 64% [28]. Van Keulen et al. demonstrated a higher sensitivity than specificity—contrary to our findings. However, more than half of the patients included in their study underwent CRC screening through the Dutch population screening program (63.5%), followed by diagnostic colonoscopy (24.2%) and surveillance colonoscopy (12.3%). This means that a substantial portion of their participants consisted of high-risk individuals (of the 447 patients, 58.8% were male with a mean age of 65 years). This composition likely led to a higher likelihood of true positives (correctly identified cases of CRC) being detected by their model, resulting in a high sensitivity score.

On the other hand, in the present study, the participant composition included two groups: one group with morphologically confirmed colorectal carcinoma and a second group of control individuals attending colonoscopy for various medical reasons, not exclusively as part of a screening program. This may mean that a proportion of the control group does not have CRC or precursor lesions, leading to fewer false positive cases and higher specificity.

In an earlier study by Amal et al., sensor analysis exhibited the ability to differentiate CRC from the control group with a sensitivity of 85%, specificity of 94%, and an overall accuracy of 91%. While this outcome holds promise, it is worth noting that the study’s CRC cohort comprised only 65 patients, and nearly a third of them were in advanced stages [27]. In contrast, our study does not possess such specific data, leaving us unaware of the proportions of early-stage and late-stage cancer patients in our cohort.

Van de Goor et al. demonstrated even more favorable outcomes through the utilization of e-nose technology to distinguish among head and neck, bladder, and colon carcinomas. This led to an 88% sensitivity and 79% specificity, resulting in an overall accuracy of 84% (MCC: 0.69) for discriminating between colon carcinoma and bladder carcinoma. Furthermore, the study yielded a sensitivity of 79% and specificity of 81%, with an overall accuracy of 81% (MCC: 0.56) for differentiating between colon cancer and HNSCC [34]. However, it should be noted that that study involved only 28 CRC patients and lacked healthy controls for comparison.

The study by Steenhuis et al. went a step further by employing an e-nose sensor to analyze the exhaled breath of individuals who had undergone curative treatment for CRC within the past 5 years and distinguishing those who subsequently developed metastatic CRC. The e-nose demonstrated the capability to detect extraluminal local recurrences or metastases of CRC with a sensitivity of 0.88 (CI 0.69–0.97) and a specificity of 0.75 (CI 0.57–0.87), ultimately achieving an overall accuracy rate of 0.81 [29]. Unlike the previous study, healthy controls were included; however, the sample size of metastatic CRC patients still remained comparably small. 

## 5. Conclusions

The results obtained during the present study confirm the potential of e-nose-based breath analyzers as a promising tool for identifying and categorizing CRC within a point-of-care clinical context. The combination of MOX sensors provided breath fingerprint patterns distinguishing healthy from diseased breath samples. Its capacity for rapid, non-invasive, and targeted CRC detection suggests encouraging prospects for future clinical applications, e.g., preoperative and postoperative patients for monitoring treatment, CRC-population-based screening, and detection of other cancers. It is plausible that a proficient screening tool could potentially identify premalignant conditions (like dysplasia); however, this goal will necessitate additional studies in future. Finally, extending the capabilities of e-nose systems by augmenting with orthogonal sensing technologies such as but not limited to infrared spectroscopy and ion mobility spectrometry will lead to modular breath diagnostic devices with improved VOC fingerprint pattern recognition for a wide variety of clinical screening scenarios [35].

## Figures and Tables

**Table 1:** Clinical features of study subjects.

Gender			Median Age	Colorectal Cancer Group		Non-Cancer Group	
	n	%		n	%	n	%
Males	113	39	64	57	54	56	30
Females	178	61	63	48	46	130	70
Total	291	100	63	105	100	186	100

n—number of participants.

**Table 2:** Classification results in the initial data set.

Classification Method	Overall Accuracy	Sensitivity	Specificity	AUC ROC
C4.5	60.9%	46.7%	68.4%	0.567
Naïve Bayes (NB)	47.1%	86.7%	26.3%	0.593
Artificial Neural Networks (ANNs)	64.4%	46.7%	73.7%	0.584
Random Forest (RF)	75.9%	43.3%	93.0%	0.684

AUC—area under the curve, ROC—receiver operating characteristic curve.

**Table 3:** Classification results in the balanced data set.

Classification Method	Overall Accuracy	Sensitivity	Specificity	AUC ROC
C4.5	65.1%	65.7%	64.3%	0.657
Naïve Bayes (NB)	60.3%	94.3%	17.9%	0.627
Artificial Neural Networks (ANNs)	66.7%	57.1%	78.6%	0.713
Random Forest (RF)	60.3%	48.6%	75.0%	0.658

AUC—area under the curve, ROC—receiver operating characteristic curve.

**Table 4:** The best result for each classification method in sensor subsets (GNP or MOX).

Classification Method	Number of Features	Overall Accuracy	Sensitivity	Specificity	AUC ROC	Feature Selection
C4.5	9	77.0%	63.3%	84.2%	0.759	MOX, Greedy sel.
Naïve Bayes (NB)	4	71.59%	34.3%	96.2%	0.671	GNP, Greedy sel.
Artificial Neural Networks (ANN)	52	72.4%	46.7%	86.0%	0.705	MOX, Evolutionary
Random Forest (RF)	8	72.4%	56.7%	80.7%	0.685	MOX, Greedy sel.

AUC—area under the curve, ROC—receiver operating characteristic curve, MOX—metal oxide sensors, GNP—gold nanoparticle sensors.

**Table 5:** The best result for each classification method.

Classification Method	Number of Features	Overall Accuracy	Sensitivity	Specificity	AUC ROC	Feature Selection
C4.5	9	77.0%	63.3%	84.2%	0.759	MOX, Greedy sel.
Naïve Bayes (NB)	1	72.4%	40.0%	89.5%	0.711	All, Greedy sel.
Neural Networks (NNs)	5	78.2%	43.3%	96.5%	0.735	All, Greedy sel.
Random Forest (RF)	75	79.3%	53.3%	93.0%	0.734	All, Evolutionary

AUC—area under the curve, ROC—receiver operating characteristic curve, MOX—metal oxide sensors.

## Data Availability

The data presented in this study are available upon request from the corresponding authors.

## Supplementary Materials

The following supporting information can be downloaded at https://www.mdpi.com/article/10.3390/diagnostics13213355/s1. Table S1. Classification results in feature subset of all data sets, feature selection using greedy forward selection; Table S2. Classification results in feature subset of all data sets, feature selection using Evolutionary Search; Table S3. Classification in the full GNP sensor set; Table S4. Classification in the full MOX sensor set; Table S5. Classification in the GNP sensor subset, Greedy stepwise selection; Table S6. Classification in the GNP sensor subset, Evolutionary Search; Table S7. Classification in the MOX sensor subset, Greedy stepwise selection; Table S8. Classification in the MOX sensor subset, Evolutionary Search; Figure S1. ROC curves of the best final classifiers: (a) C4.5 (MOX sensors, greedy selection of features), (b) Naïve Bayes (all sensors, greedy selection of features), (c) Neural Network (all sensors, greedy selection of features), (d) Random Forest (all, Evolutionary Search for Features).
