# Supplementary material for: The Detection of Colorectal Cancer through Machine Learning-Based Breath Sensor Analysis
Source: Diagnostics (Basel). 2023 Oct 31;13(21):3355. doi: 10.3390/diagnostics13213355 (PMC10648537; doi:10.3390/diagnostics13213355)
Supplement: Supplementary file 1 [file diagnostics-13-03355-s001.zip › diagnostics-2641846-supplementary.pdf]

Supplementary Materials

Table S1. Classification results in feature subset of all data set, feature selection using greedy forward selection.

Classification method	Number of features	Overall Accuracy	Sensitivity	Specificity	AUC ROC
C4.5	3	75.9%	40.0%	94.7%	0.674
Naive Bayes	4	72.4%	40.0%	89.5%	0.711
Neural Networks	5	78.2%	43.3%	96.5%	0.735
Random Forest	6	73.6%	53.3%	84.2%	0.760

Table S2. Classification results in feature subset of all data set, feature selection using Evolutionary Search.

Classification method	Number of features	Overall Accuracy	Sensitivity	Specificity	AUC ROC
C4.5	54	72.4%	53.3%	82.5%	0.654
Naive Bayes	164	48.3%	83.3%	29.8%	0.586
Neural Networks	73	64.4%	43.3%	75.4%	0.685
Random Forest	75	79.3%	53.3%	93.0%	0.734

Table S3. Classification in the full GNP sensor set.

Classification method	Number of features	Overall Accuracy	Sensitivity	Specificity	AUC ROC
C4.5	145	67.0%	40.0%	84.9%	0.566
Naive Bayes	145	59.1%	45.7%	67.9%	0.637
Neural Networks	145	60.2%	42.9%	71.7%	0.603
Random Forest	145	69.3%	34.3%	92.5%	0.727

Table S4. Classification in the full MOX sensor set.

Classification method	Number of features	Overall Accuracy	Sensitivity	Specificity	AUC ROC
C4.5	135	49.4%	60.0%	43.9%	0.532
Naive Bayes	135	39.1%	70.0%	22.8%	0.500
Neural Networks	135	65.5%	43.3%	77.2%	0.616
Random Forest	135	74.7%	50.0%	87.7%	0.655

Table S5. Classification in the GNP sensor subset, Greedy stepwise selection.

Classification method	Number of features	Overall Accuracy	Sensitivity	Specificity	AUC ROC
C4.5	5	63.64%	31.4%	84.9%	0.641
Naive Bayes	4	71.59%	34.3%	96.2%	0.671
Neural Networks	5	67.0%	20.0%	98.1%	0.673
Random Forest	7	68.2%	45.7%	83.0%	0.779

Table S6. Classification in the GNP sensor subset, Evolutionary Search.

Classification method	Number of features	Overall Accuracy	Sensitivity	Specificity	AUC ROC
C4.5	49	60.2%	45.7%	69.8%	0.570
Naive Bayes	47	63.6%	25.7%	88.7%	0.653
Neural Networks	30	62.5%	68.6%	58.5%	0.668
Random Forest	30	72.7%	40.0%	94.3%	0.770

Table S7. Classification in the MOX sensor subset, Greedy stepwise selection.

Classification method	Number of features	Overall Accuracy	Sensitivity	Specificity	AUC ROC
C4.5	9	77.0%	63.3%	84.2%	0.759
Naive Bayes	3	66.7%	13.3%	94.7%	0.565
Neural Networks	7	62.1%	40.0%	73.7%	0.582
Random Forest	8	72.4%	56.7%	80.7%	0.685

Table S8. Classification in the MOX sensor subset, Evolutionary Search.

Classification method	Number of features	Overall Accuracy	Sensitivity	Specificity	AUC ROC
C4.5	43	60.9%	63.3%	59.6%	0.606
Naive Bayes	14	63.2%	13.3%	89.5%	0.579
Neural Networks	52	72.4%	46.7%	86.0%	0.705
Random Forest	57	73.6%	50.0%	86.0%	0.649

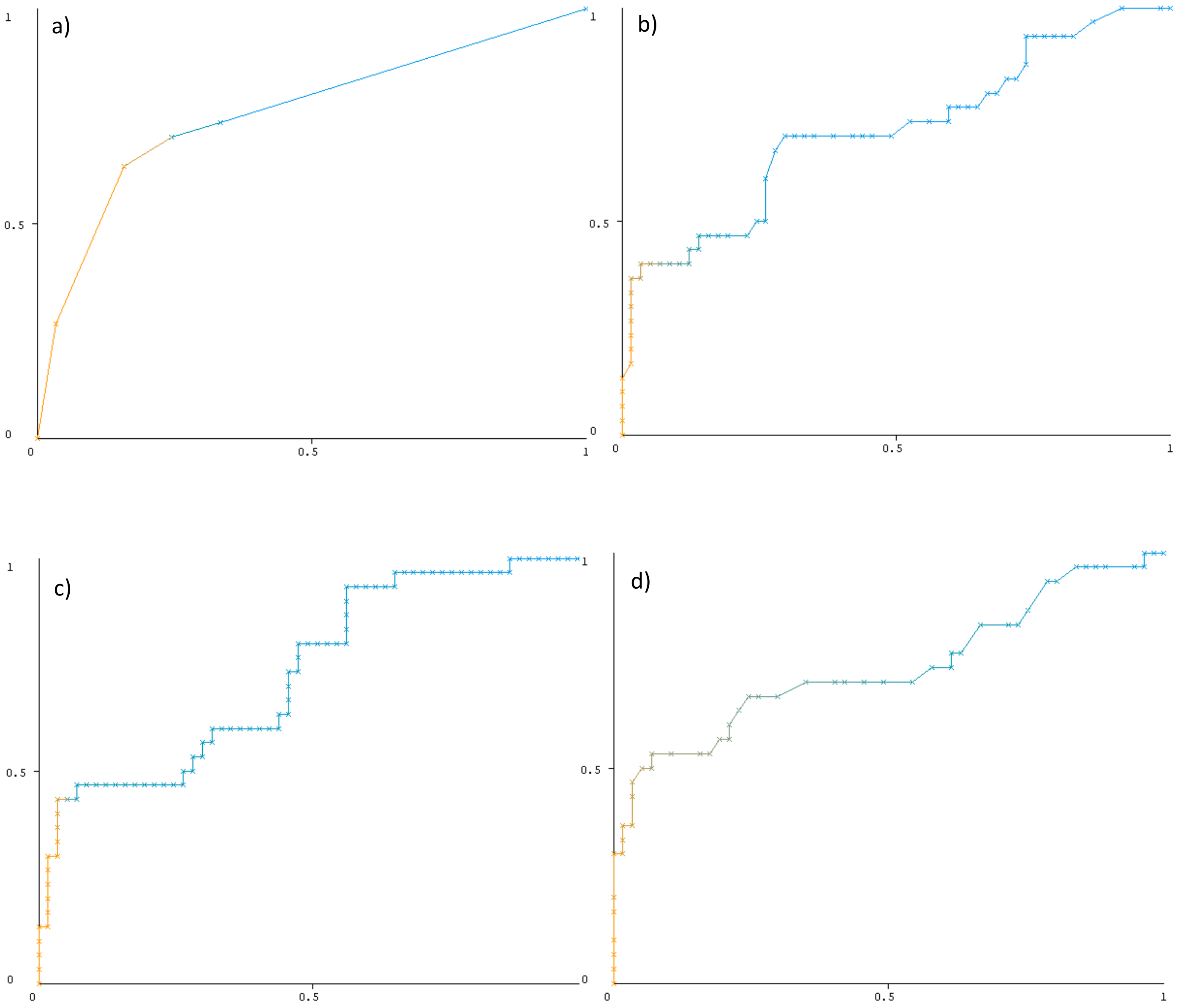

Figure S1. ROC curves of the best final classifiers: a) C4.5 (MOX sensors, greedy selection of features), b) Naïve Bayes (all sensors, greedy selection of features, c) Neural Network (all sensors, greedy selection of features), d) Random Forest (all, evolutionary search for features)
